# Functional Silane-Based Nanohybrid Materials for the Development of Hydrophobic and Water-Based Stain Resistant Cotton Fabrics Coatings

**DOI:** 10.3390/nano12193404

**Published:** 2022-09-28

**Authors:** Silvia Sfameni, Tim Lawnick, Giulia Rando, Annamaria Visco, Torsten Textor, Maria Rosaria Plutino

**Affiliations:** 1 Department of Engineering, University of Messina, Contrada di Dio, S. Agata, 98166 Messina, Italy; 2 Institute for the Study of Nanostructured Materials, ISMN–CNR, Palermo, c/o Department ChiBioFarAm, University of Messina, Viale F. Stagno d’Alcontres 31, 98166 Messina, Italy; 3 TEXOVERSUM School of Textiles, Reutlingen University, 72762 Reutlingen, Germany; 4 Department of ChiBioFarAm, University of Messina, Viale F. Stagno d’Alcontres 31, Vill. S. Agata, 98166 Messina, Italy; 5 Institute for Polymers, Composites and Biomaterials CNR IPCB, Via Paolo Gaifami 18, 95126 Catania, Italy

**Keywords:** sol–gel, (3-Glycidyloxypropyl)trimethoxy silane, functional cotton fabrics, hydrophobicity, nanohybrid coatings

## Abstract

The textile-finishing industry, is one of the main sources of persistent organic pollutants in water; in this regard, it is necessary to develop and employ new sustainable approaches for fabric finishing and treatment. This research study shows the development of an efficient and eco-friendly procedure to form highly hydrophobic surfaces on cotton fabrics using different modified silica sols. In particular, the formation of highly hydrophobic surfaces on cotton fabrics was studied by using a two-step treatment procedure, i.e., first applying a hybrid silica sol obtained by hydrolysis and subsequent condensation of (3-Glycidyloxypropyl)trimethoxy silane with different alkyl(trialkoxy)silane under acid conditions, and then applying hydrolyzed hexadecyltrimethoxysilane on the treated fabrics to further improve the fabrics’ hydrophobicity. The treated cotton fabrics showed excellent water repellency with a water contact angle above 150° under optimum treatment conditions. The cooperative action of rough surface structure due to the silica sol nanoparticles and the low surface energy caused by long-chain alkyl(trialkoxy)silane in the nanocomposite coating, combined with the expected roughness on microscale due to the fabrics and fiber structure, provided the treated cotton fabrics with excellent, almost super, hydrophobicity and water-based stain resistance in an eco-sustainable way.

## 1. Introduction

Textiles are critical to a country’s growth and industrialization. In recent decades, many efforts have been made to develop innovative and nanostructured surface treatments in order to modify the mechanical and surface properties of natural and synthetic fabrics [1], thus replacing commonly used hazardous chemicals with products that are respectful of the environment and of health, while maintaining functional characteristics [2,3]. New multifunctional protective and smart textiles have been developed in response to growing technical breakthroughs, new standards, and a customer demand for textiles that are not only attractive but also practical [4,5,6]. In this regard, silica-based organic-inorganic nanostructured finishes could be considered an interesting alternative [7,8].

**Figure 1:**
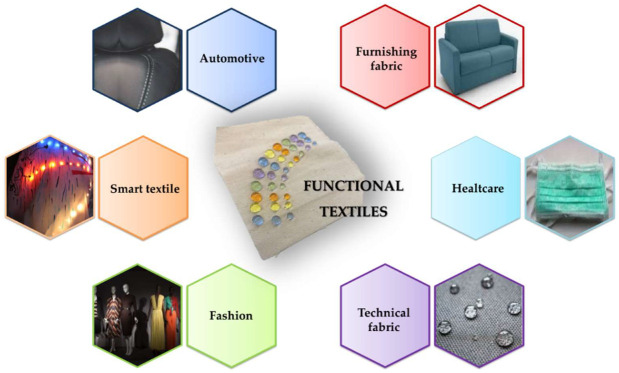
Functional and technical textiles for improved performance, protection, and health: types, application, and market.

In recent years, the sol–gel approach has shown to be a creative and efficient method of improving the characteristics of fibers [9,10,11,12,13]. This approach comprises a diverse synthetic pathway that may be used to create novel materials with high molecular homogeneity and excellent physical and chemical characteristics. Due to its biocompatibility and non-toxicity, the sol–gel technique has been used to confer several functional properties to different textiles materials [14,15,16,17], such as antimicrobial [18,19,20,21,22], self-cleaning [23], water repellency [24,25,26], flame retardancy [27,28,29], and sensing [30,31,32,33], as well as improving the dye ability of fabric samples (see Figure 1) [34].

Sol–gel synthesis and applications follow a two-step procedure based on the hydrolysis and condensation of metal or semi-metal alkoxides: after forming a hydrolyzed metal alkoxide solution at room temperature, textile materials are impregnated with the latter, and the samples are cured at a specific temperature to obtain a porous 3D fully inorganic or hybrid organic–inorganic nanostructured coating. Consequently, there are many alternatives for the formulation and application of sol–gel coatings in the field of textile functionalization, as choosing the correct and opportune functional silane precursor, which allows for the desired chemical and physical properties improvement of the fabric. Because of the moderate processing conditions required and the use of ordinary commercial textile finishing machines, in recent years, there has been a surge in interest in the application of the sol–gel approach to produce functional coated textiles [35,36,37,38,39], for example water-repellent fabrics. In general, surfaces that exhibit water contact angles > 150° (on which water drops remain almost spherical and easily roll off, also able to remove dirt particles in their path), are usually called superhydrophobic surfaces [40,41]. Superhydrophobic surfaces have recently attracted significant attention within the scientific community because of their unique water-repellent, anti-icing, anti-contamination, anti-sticking, and self-cleaning properties and their potential for practical applications [42,43].

Much of this research has been inspired by lotus leaves and has demonstrated that superhydrophobic surfaces may be produced by combining the right surface roughness and low surface free-energy [44,45,46]. The surface of lotus leaf was first examined by Barthlott in 1970 using scanning electron microscopy and it was found that the surface has small micro-protrusions covered with nano-hairs which are covered with low surface free-energy wax substances [46].

Surface roughness and surface free-energy were used to create superhydrophobic surfaces on cotton textiles. Different nanoparticles, including zinc oxide, titanium dioxide, silica nanoparticles (SNP), or alkoxysilane-based nano-sols [47] were added to cotton fabric to provide surface roughness. Fluorocarbons and silicones are examples of substances with low surface free-energy that might change the surface energy of cotton substrate [48].

In particular, fluoroalkylsilanes were used to further increase the surface water-repellency. Most recently, the ECHA’s committee (Committee for Risk Assessment—ECHA—European Union) recommended restriction for some perfluoroalkyl substances (PFAS) regarding some application fields. In particular, fluoro-chemical finishing products are banned for textile applications in all EU states, while only some exemptions (i.e., in medical, technical, and workwear textiles) are accepted, but a complete restriction is expected in EU shortly, with a movement towards more widespread use of hydrophobic alkyl silanes. Currently, there are reports of the creation of rough surface micro/nanostructures using silane nanoparticles or nano-sols [49,50,51,52] and the subsequent modification with hydrophobic materials (e.g., fluoroalkylsilane, long-chain alkyl(trialkoxy)silane) to create superhydrophobic surfaces through a multi-step procedure [53,54].

Lakshmi et al. [55] produced superhydrophobic sol–gel nanocomposite coatings by adding silica nanoparticles to an acid-catalyzed ethanol–water solution of methyltriethoxysilane (MTEOS), while Huang et al. [56] created superhydrophobic surfaces by covering a silane-based coating in ethanol with a low surface-energy material 1H, 1H, 2H, 2H-perfluorooctyltrichlorosilane. By spraying an ethanol suspension of silica sol and silica microspheres, Shang et al.’s method [57] produced superhydrophobic silica coatings that were then hydrophobically treated with a solution of 1H, 1H, 2H, 2H-perfluorodecyltriethoxysilane (PFDTS). In order to create superhydrophobic silica films, Ramezani et al. [58] examined the two-step dip coating method using a sol–gel procedure. They coated a silica-based solution, and then modified it with isooctyltrimethoxysilane as a hydrophobic agent. According to studies [59,60], fluorine-based hybrid materials are the most successful in reducing the free-energy surface. However, some of the molecules are carcinogenic, highly costly, and not environmentally friendly.

**Figure 2:**
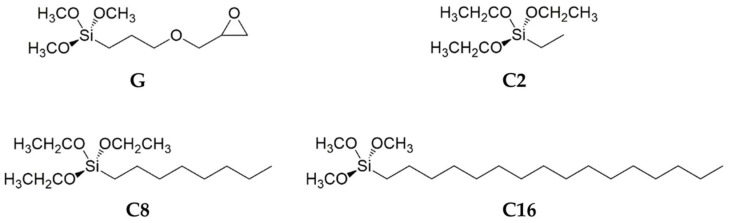
Alkyl(trialkoxy)silanes employed in this work.

In this work, co-condensation of (3-Glycidyloxypropyl)trimethoxysilane (hereafter, GPTMS or G) and different non-fluoro compounds, i.e., Hexadecyltrimethoxysilane C16, Triethoxy(octyl)silane C8 and Triethoxy(ethyl)silaneC2, as showed in Figure 2, was conducted in the presence of an acid catalyst to obtain functional nanohybrids via a one-step process.

By varying the length of the chain of the alky(trialkoxy)silane, R-Si(OR’)_3_, modified silane-based nanocomposite hydrosols, R-Si(O-)_3_, were obtained with high dispersion stability. By applying R-Si(O-)_3_ nanocomposite hydrosols to cotton fabrics, almost superhydrophobic cotton surfaces were obtained, as well as surface roughness and low surface energy. This study aimed to employ a multicoating eco-friendly technique in sol–gel textile finishing by examining the impact of various alkyl(trialkoxy) silane precursors on the silica-based mesh and, finally, to study the implemented mechanical characteristics of the treated cotton fabric. GPTMS is a useful molecule capable of forming extensive cross-links between the silanol groups of the polyoxysilane matrix and promoting adhesion through the opening of the epoxy ring on the treated polymers. It is a silica precursor that is frequently used for silica-based hybrid textile finishing [61,62].

The characteristics and the bi-functionality of the GPTMS, as well as its potential as a new textile finishing agent, should be investigated because there has not yet been much research on the impact of GPTMS synthetic parameters on the mechanical properties of fabrics made with both natural and synthetic polymers [63,64]. Indeed, because the chemical structure of fabric substrates is significant for the stability of the applied coatings, which is dependent on the thermodynamic affinity between the silica precursor and the selected textile samples, natural cotton textiles were employed in the current investigation.

Cotton fabrics were chosen as model substrates owing to their unique properties such as high hydroxyl group content, hydrophilic nature, and broad use, which allows them to be used not only in fabrics and garments but also in technical or smart textiles. Moreover, the use of alkyl(trialkoxy)silane has numerous advantages; it is low-cost and once polymerized is a non-toxic material [65,66,67,68], and a promising alternative for achieving durable hydrophobic fabrics. The final goal of this work was to illustrate an easy, environmentally friendly, and adaptable technique for generating hybrid coatings that are compatible with cellulose fabrics and their physical intrinsic features so that they can find applications in different sectors such as textiles [69], biomedical [70], furnishings [71], environmental remediation [72] and sensing [73]. The hydrophobicity was evaluated by WCA and WSA measurements.

By the characterization methods, the morphological qualities, surface chemistry, and durability of the sol–gel coatings were mainly evaluated using optical microscopy and SEM, comparing treated and untreated cotton textiles as a reference. Moreover, the water based anti-stain performances of the treated fabrics and, qualitatively, their oil–water separation ability towards paraffin oil were evaluated. In fact, functionalizing textiles with coatings based on the use of GPTMS in conjunction with functional alkyl(trialkoxy)silane could result in useful multifunctional nanocomposites for potential applications in the field of advanced, environmentally friendly nanohybrid materials, which would then find use in numerous nanotechnology fields.

## 2. Materials and Methods

### 2.1. Fabric

Knitted pure cotton fabric 100% (scoured and bleached, 1.4 g/cm^2^ or 0.014 g/cm^2^ and 0.2 mm thick) was used as natural fabric and it was provided by the School of Textile and Design (University of Reutlingen, Germany).

### 2.2. Chemicals

The (3-Glycidyloxypropyl)trimethoxysilane (G),Triethoxy(ethyl)silane (C2), Triethoxy(octyl)silane (C8) and Hexadecyltrimethoxysilane (C16), were all purchased at the highest purity level and used as received from Sigma Aldrich (Merk GaA, Darmstadt, Germany), without any further purification. Hydrochloric acid HCl 37% was used as sol–gel catalyst. Ethanol 96% vol. was purchased from Sigma Aldrich and used as solvent.

### 2.3. Preparation of the Nanosol Solution

The sol–gel solution was prepared by mixing the G precursor in combination with an equimolar amount of each of the three different alkoxysilanes featuring increasing length of the hydrocarbon chain (namely, C2, C8, C16). The obtained mixture was stirred while ethanol was added slowly at room temperature. Ethanol was used as dilution medium while HCl was added dropwise to induce the hydrolysis–condensation reaction. The resulting mixture was vigorously stirred at room temperature for 24 h.

### 2.4. Sol–Gel Treatment of Cotton Fabrics

**Figure 3:**
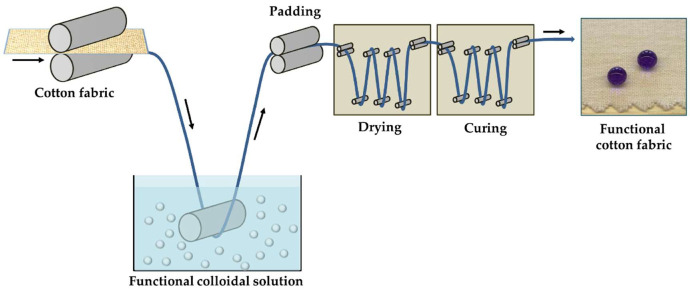
Pad-dry-cure process employed for finishing cotton fabrics.

Cotton fabrics were cut into square pieces (10 × 15 cm), weighted and then impregnated with the solution using the dip-pad-dry-cure method (Figure 3).

First, the cotton fabric samples were immersed in the solutions for 5 min at room temperature before being washed with water. Second, an automated padder (simple two roller lab-padder of Mathis, Oberhasli, Switzerland) with a nip pressure of 2 kg/cm^2^, was used to pad the cotton fabric samples. They were then dried at 80 °C for 6 min.

**Figure 4:**
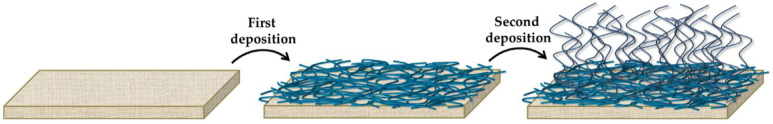
Double sol–gel-based coating application for the development of the treated functional cotton fabrics.

The process was repeated three times. In addition, samples of cotton were dipped in the alkyl(trialkoxy)silane-based ethanol solution (1.0 g, 30 mL) for 5 min, giving rise to a double-coating deposition (Figure 4).

The impregnated fabrics were finally put in the oven support and dried to a constant weight in the oven at 130 °C for 6 min: during this time the evaporation of water and ethanol and the sol–gel reactions took place.

**Figure 5:**
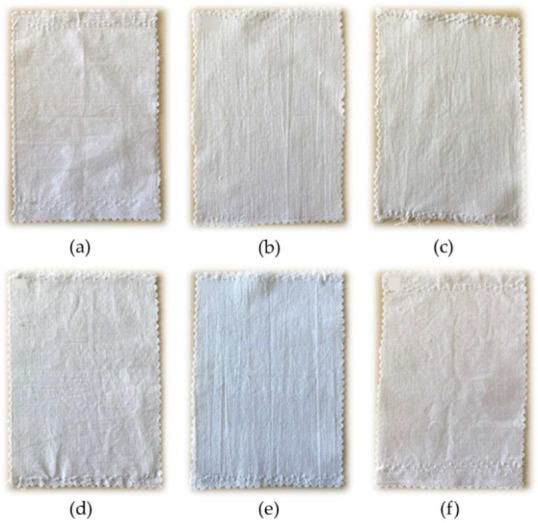
Cotton modified with sol–gel solution. (**a**) COT + G, (**b**) COT + G/C2_C′2, (**c**) COT + G/C8_C′8, (**d**) COT + G/C16_C′16, (**e**) COT + G/C2_C′16, and (**f**) COT + G/C8_C′16.

This was confirmed by the color change of the fabrics as shown in Figure 5 and then modified cotton fabric was weighted, after being climatized for 24 h in a standard climate chamber. The composition of the functional nanohybrid sols employed for the double deposition process is shown in Table 1.

Subsequently, total dry-solid add-ons on the cotton samples (the weight gain, A wt. %) was determined by weighing each sample before (W_i_) and after the impregnation with the solution and the subsequent thermal treatment (W_f_) (Table 2).

The weight gain of the treated fabric was calculated using the following formula:

(1) $$A=\frac{W_{f\;}-{\;W}_{i}}{W_{f}}\;\times \;100$$

### 2.5. Characterization and Functional Properties of Treated Fabrics

*Wettability.* Aqueous liquid repellency: water/alcohol solution tests were performed using a test reagent formulated using the AATCC test method 193-2007 Aqueous Liquid Repellency: Water/Alcohol Solution Resistance Test. Using a 5 μL water droplet at room temperature, the sessile drop technique (according to the international standard ASTM D7334) was used to measure the static water contact angles (WCA). One representative WCA was generated by averaging ten readings. The AATCC Test Method 22-2005, which is applicable to any textile fabric, was used to conduct the spray testing in order to examine the dynamic wettability of the treated samples. Three fabric samples, measuring 150 mm × 150 mm, are required to obtain one representative value for the spray testing. The tester’s funnel is filled with 250 mL of distilled water, which is then sprayed onto a sample of cotton at a 45° angle. Three knocks are applied before the sample is removed. The water repellency rating (WRR) is used to examine the extent of the wetting. Valuation is carried out by comparing the wetted sample’s appearance with the wetted pristine cotton sample used as standard. Better hydrophobicity is indicated by a higher rating. The maximum and minimum ratings are 100 and 0, respectively.

*Optical microscopy.* Optical images were recorded by means of a Hirox digital microscope, model KH8700 (Hirox, Tokyo, Japan) by mounting a MX(G)-5040Z lens at room temperature.

*Scanning Electron Microscopy (SEM) Analysis.* The two-dimensional morphology and structure of the surface fibers of the of the original and treated cotton fabrics were observed at 2.0 kV using scanning electron microscope (SEM, SU-70, Hitachi, Chiyoda, Tokyo, Japan) and a magnification of 1000 × and 4000 × for the insets. All the samples were sputter-coated with Aurum prior to testing.

*Self-cleaning ability.* To evaluate the wetting behavior, several liquids including coffee, milk, tea, methylene-blue-dyed water, pH = 1 acid (HCl), pH = 14 alkali (NaOH), and salt solution (NaCl) were individually placed onto the GC16_C’16-modified cotton fibers. In order to test the self-cleaning abilities, soil was applied to the surface of the modified cotton fibers and washed with blue-dyed water.

*Oil/water separation ability*. The oil/water separation capabilities of the modified cotton textiles were tested using paraffin oil. The paraffin oil was colored using the coloring agent oil red before to the oil/water separation experiments.

*Moisture analysis.* The moisture-transfer properties of all the cotton fabrics samples were evaluated by using the KERN DBS moisture meter (KERN & SOHN GmbH-TYPE DBS60-3) that often replaces others drying processes, such as the laboratory dryer, because it allows for shorter measurement times. The moisture-transfer properties of all the cotton fabrics samples were evaluated through the principle of thermogravimetry. In this method, to determine the difference in moisture in a material, the sample is weighed before and after drying. In the case of the KERN DBS moisture meter, the radiation penetrates the sample and is transformed into thermal energy, heating up from the inside out. A small amount of radiation is reflected by the sample and this reflection is larger in dark samples than in light ones. Therefore, light samples, such as cotton in this case, reflect more thermal radiation than dark ones and therefore require a higher drying temperature, which is why a drying temperature of 130 °C is used for the analysis. Moisture measurement protocol (unit indicating the result: M/W, drying mode: TIME, drying temperature: 130 °C). The hygroscopicity ratio was calculated by Equation (2), which was used to as an indicator for evaluating the hygroscopicity of these cotton fabrics.

(2) $$\mathrm{Hygroscopicity}\;\mathrm{ratio}\left(\%\right)=\frac{m_{2}-m_{1}}{m_{1}}\times 100\%$$

where, m_2_ is the weight of the conditioned sample and m_1_ is the initial weight of original samples.

*Air-permeability test.* The air permeability of treated fabrics, which serves as an indication of their breathability, was investigated. The permeability of the samples was measured by the use of an apparatus (FX3300, Tex Test AG, Schwerzenbach, Switzland) under the air pressure of 125 Pa, according to the ASTM D737-96 standard test method.

## 3. Results

### 3.1. Nanosol Synthesis and Application on Cotton Fabrics

**Figure 6:**
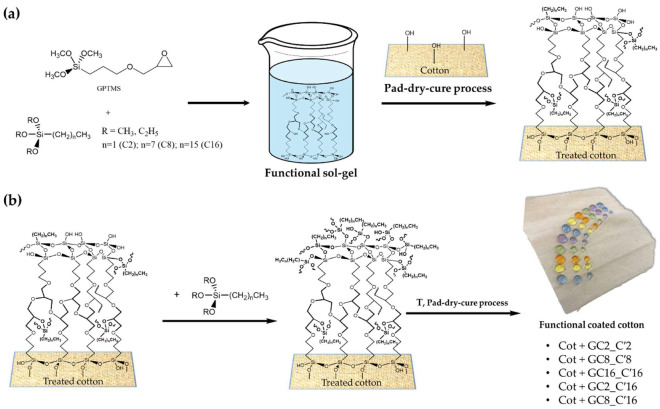
Two-step synthetic pathways for the development of the final functional coated cotton fabrics involving the condensation reaction between the cotton cellulose and alkoxysilane ends (**a**), and subsequent anchorage of alkyl(trialkoxy)silanes (**b**).

The sol–gel technique is a very versatile method leading to the formation of different kind of interesting functional nano- and micro-structured materials, with a fine control and tuning of their surface chemistry and of the bulk nanocomposite/nanohybrid properties of the end-products. In this study, the sol–gel synthesis and application followed a two-step pathway (Figure 6a,b) to finally yield the desired hydrophobic cotton fabrics.

In the first step, the functional sol was prepared by reaction of the bifunctional GPTMS alkoxysilane and either the C2, or C8, or C16 alkyl(trialkoxy)silane, respectively, featuring different length alkyl chains. As previously reported [63,64], the functional nanosol solution is obtained by subsequent hydrolysis and condensation reaction, thus producing colloidal particles or dissolved pre-condensed polymeric hybrid polymers. Once applied on cotton fabrics and with additional heat treatment at higher temperatures by a pad-dry-cure process, the gel will give rise to a functional xerogel.

In order to form a more efficient hydrophobic coated cotton, it seemed to be worthwhile to use a second pad-dry-cure step by employing C2, or C8, or C16 alkyl(trialkoxy)silanes, respectively, thus giving rise to five functional treated cotton samples, namely Cot + GC2_ C′2, Cot + GC8_ C′8, Cot + GC16_ C′16, Cot + GC2_ C′16, Cot + GC8_ C′16; Cot + G that was used as cotton fabric reference in all experimental measurements.

### 3.2. Wettability Measurement

Water contact angle (WCA) measurement was used to explore the static hydrophobicity of the treated cotton samples, and the spraying test was used to evaluate the dynamic water repellence.

#### 3.2.1. Aqueous Liquid Repellency: Water/Alcohol Solution Test

To evaluate the level of anti-wettability or repellency, the contact angles of liquids with different surface tensions was measured, using a test reagent formulated using the AATCC test method 193-2007 Aqueous Liquid Repellency [74]. The aqueous-liquid repellency test (also known as the water-rating method WRA) describes a procedure whereby 20 μL drops of solution with increasing concentration of isopropyl alcohol is placed onto the fabric. If the drops of a solution do not wet the textile within 10 s, the next solution with a higher share of isopropanol is applied. The rating number is assigned based on the solution with the highest isopropanol share that does not wet the textile within the 10 s.

Table 3 outlines which concentration of isopropyl alcohol solution equates to which rating number.

The fabric must be able to repel the solution for 10 s to be deemed successful. As a static test, this method could be considered to be more stringent than other water-drop methods as it makes use of solutions with surface tensions lower than that of water.

#### 3.2.2. Sessile Drop Method

**Figure 7:**
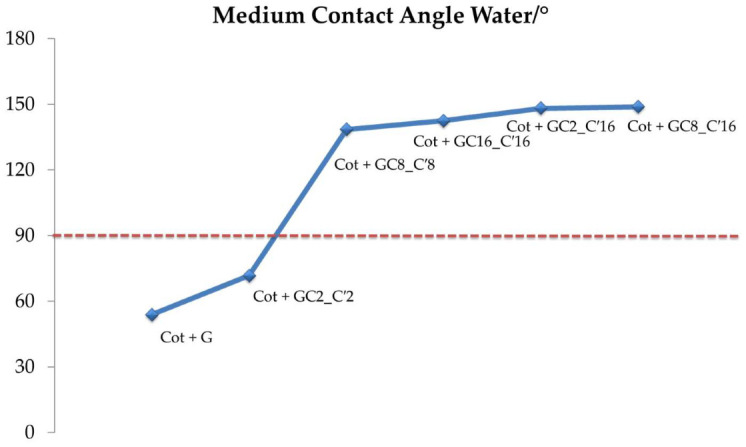
Schematic graph representative of the static water-contact angles of Wenzel θw values. The line between the data points is only a guide for the eye.

**Figure 8:**
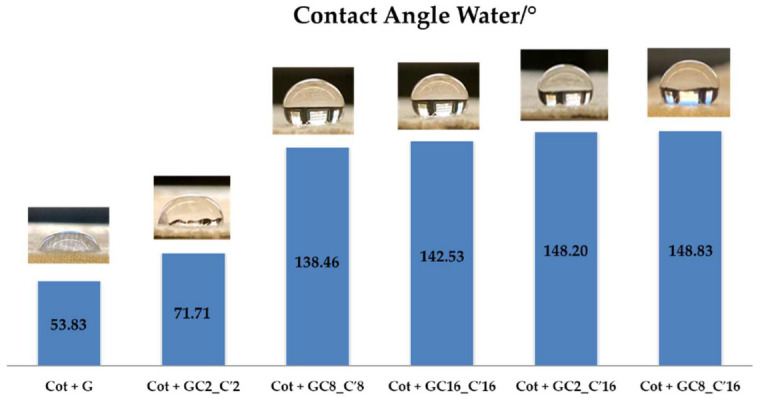
Histograms of the contact angle θw of the cotton fabrics treated by the nanocomposite sol samples G, GC2_C′2, GC8_C′8, GC16_C′16, GC2_C′16, and GC8_C′16 with photos of the representative drops.

As a way to examine the effect of the length of the hydrocarbon chains and their distribution/orientation into the nano composite sol on the hydrophobicity of the treated cotton fabric, the cotton fabrics samples were coated by using three different sols, as prepared by changing the length of the functional alkyl(trialkoxy)silane, from 2 to 8 to 16 methylene groups. In a typical process, a deionized water droplet (ca. 5 µL) was dropped carefully onto the surface at ambient temperature and the images were captured using the accessory digital camera. All the water contact-angle values reported herein were obtained as averages of five measurements performed on different points of the sample surface so as to improve the accuracy [75,76,77]. The results were shown in Figure 7 and Figure 8.

WCAs for the treated cotton fabrics ranged from 71.71° to 142.53°. In particular, the cotton fabric coated by the nanocomposite hydrosol with the shortest hydrocarbon chain (GC2_C′2) displayed the poor hydrophobicity, showing the lowest WCA of 71.71°. However, the cotton fabric that had been treated with the nanocomposite sol bearing the longest hydrocarbon chain (GC16_C′16) demonstrated exceptional hydrophobicity with the maximum WCA of 142.53°. In this regard, it was shown that nanocomposite sols, treated with mixed alkyl(trialkoxy)silane (GC2_C′16 and GC8_C′16), are beneficial for the formation of a brush effect surface topography [67] on the coated fabrics, thus resulting in an improvement of the hydrophobicity of treated fabrics of 148.20° and 148.83°, respectively.

Table 4 and Figure 8 indicate the hydrophobicity of the treated cotton samples.

According to Figure 7, the WCA of the treated cotton increased as well as the length of the alkyl chain. The final treatment with HDTMS-based sol led to a lowering of the surface energy of the cotton fabric, with a consequent improvement in hydrophobicity. As indicated in Figure 8, the WCA of all cotton fabric samples was less than 150°. Although a water droplet can sit on the surface of the coated cotton cloth, it does not achieve super-hydrophobicity.

**Figure 9:**
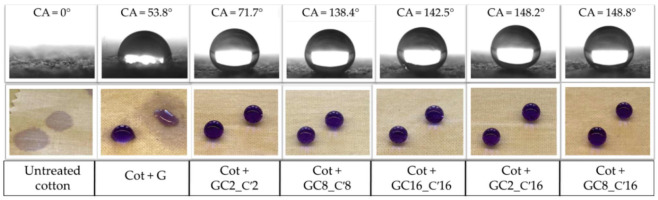
The images show the change in water contact angle and colored water droplets sitting on coated cotton fabrics and untreated cotton.

Furthermore, as shown in Figure 9, when we exposed the coated cotton textile to water droplets, the fabric successfully showed hydrophobicity similar to that present in the rose petals. The double-coating synthetic method used to improve the cotton surface hydrophobicity by pad-dry-cure deposition of the prepared nanohybrid coatings, as demonstrated using water contact angle, was shown to be successful. As a matter of fact, while the control sample had a water contact angle of 53.8°, with the incorporation of hydrophobic long-alkyl-chain silane coupling agent onto surface of cotton fabrics, the water contact angle for the Cot + GC8_C′16 nanohybrid increased to 148.83°. These values are generally higher (ca. 30–40°) than those obtained with a “grafting to” chemisorption method of the corresponding GC* functional sol–gel, as recorded on a glass slide coated after an opportune commercial primer and tie-coat layer [67].

**Figure 10:**
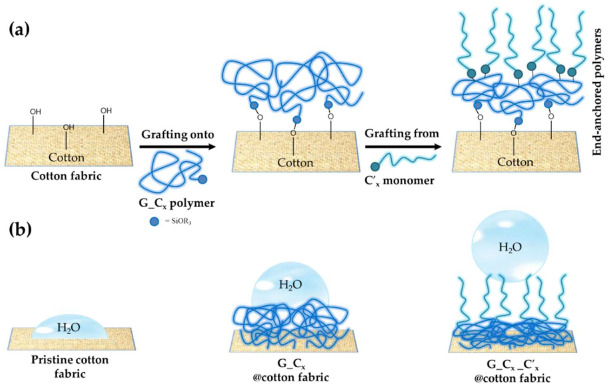
Double surface functionalization of cotton fabrics with alkyl(trialkoxy)silane polymer shell by “grafting to” or “grafting from” covalent grafting techniques (**a**) and corresponding observed surface hydrophobicity of the coated cotton fabrics (**b**).

In this study the key step was shown to be the second coating application of the bulk functional C2, C8, and C16 silanes, especially those featuring increase of the length of the alkyl(trialkoxy) chain (i.e., C8 and C16), by the “grafting from” chemisorption covalent technique [78,79,80] (Figure 10a). The treated cotton fabrics exploited the observed surface hydrophobicity, as shown in Figure 10b.

In this process, on the first coated cotton surface, characterized by a silane-based 3D polymeric matrix obtained through a “grafting onto” procedure, a second functional coating was deposited, and at the end a polymerization process under conventional dry-cure conditions occurred. This latter “grafting from” process could bring a better control of the in-situ grafting density, composition, and molar ratio of the polymer brush shells. It is also not limited by the steric hindrance of the incoming functional alkyl chains, thus resulting significantly in lower surface energy and higher hydrophobicity [81] (Figure 10b).

Moreover, it has been reported that improvement of the properties, morphological characteristics, and fiber roughness of different functional coatings on textile fabrics, leading to enhanced mechanical and hydrophobic behaviors, can be achieved by employing a double-layer deposition approach [82,83,84].

#### 3.2.3. Spray Test

Spray testing (AATCC 22-2005) was also used to study the dynamic wettability of the treated cotton fabric [85]. Spray testing quantifies the degree of wetness when the fabric is sprayed with water. The water-stain characteristics at different wetting degrees (in ISO standard ratings) are listed in Table 5. The wettability level of the uncoated fabric was 0, referring to no water repellency. After one coating of the sample COT + G, the wettability level was found to be 0, which was similar to uncoated fabric. However, the wettability level of the coated fabrics increased up to 50 after two coatings and remained at the same value after three coatings. This result indicates that the water repellent property of the fabrics could be enhanced by increasing the number of depositions.

According to the spray test, the treated cotton fabric samples COT + GC8_C′8 and COT + GC16_C′16 had a rating number corresponding to 100. The 250 mL of water in contact with the treated cotton immediately slipped away from the fabric, leaving only a few drops of water attached. In the light of the obtained results, cotton fabrics possess excellent hydrophobicity with a low surface energy. In contrast, when the cotton fabric was coated by the GPTMS-based sol without alkysilane modification, its surface shows higher surface energy. In addition, when a water droplet was applied on the pristine cotton fabric, it quickly spread.

#### 3.2.4. Self-Cleaning Ability Measurement

**Figure 11:**
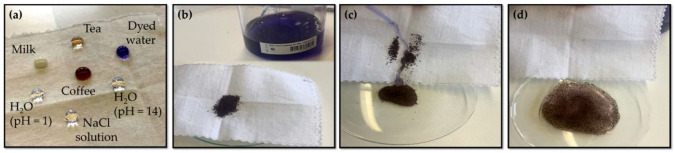
Photograph demonstrating the almost super-hydrophobicity of GC16_C′16-modified cotton textiles against various liquids (**a**) and real-time photographs demonstrating their water-based stain resistance performance (**b**–**d**).

The self-cleaning capabilities of the GC16_C′16-modified cotton textiles are displayed in Figure 11. The GC16_C′16-modified cotton fabrics, as shown in Figure 11a, exhibited excellent almost super hydrophobicity against blue-dyed water, strong acid (HCl, pH = 1), strong alkali (NaOH, pH = 14), and salt solution (NaCl 0.9 wt.%, pH = 7). They also exhibited excellent, almost super, hydrophobicity against milk, coffee, and tea.

It can be also seen that different liquids all exist in a similar spherical shape and this indicates that the coating has a wide range of adaptability in practical applications and implying that they may be flexible to self-cleaning under a variety of settings. Moreover, it can be clearly observed that the dust on the surface of the coating was completely removed after washing with water. As a matter of fact, water could readily flow over the surface of the sample and remove dust, as illustrated in Figure 11b–d, indicating that GC16_C’16 coating, in particular, has a very good water-based stain resistance.

It is assumed that the hydrophobic properties of the coating may be principally due to the air trapped in the nanoscale gaps of the almost super hydrophobic surface, which decreases the area of interaction between the soil and the coating and assists soil to roll-off from its surface [86,87].

### 3.3. Oil/Water Separation Ability

It is well known that cotton fabric is capable of absorbing large amounts of water due to its high hydrophilicity [88]. The above-mentioned, GC8_C′8, GC16_C′16, GC2_C′16, and GC8_C′16 coatings endowed the cotton fabrics with almost super hydrophobicity. In contrast, the modified cotton fabrics also have reduced surface energy that allows a repelling of water, but wetting with oils is still possible—that means the surfaces can be able to separate water from oil [89].

**Figure 12:**
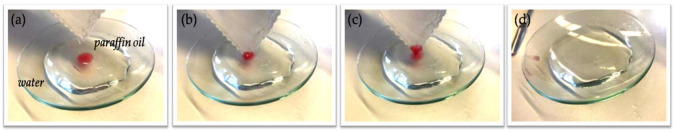
Real-time photos demonstrating the removal of paraffin oil (**a**–**d**) droplets from water using GC16_ C′16 modified cotton fibers.

Figure 12 shows a sequence of images in which a cotton fabric treated with the GC16_C’16 sol was used to qualitatively study its oil/water separation ability by using paraffin oil as a model oil [90,91]. Paraffin oil was further dyed red for better observation [92]. Therefore, a droplet of red-tinted paraffin oil (with a density lower than that of water) was located in the watch glass, as illustrated in Figure 12a–d. A piece of treated fabric was soaked in the oil/water mixture to make it completely contact with the oil. It can be clearly observed that oil droplets quickly spread and even permeated into fabric, indicating the lipophilic nature of the coating. This selective adsorption is indicative of remarkable hydrophobicity or oleophilicity of the sample [93].

In particular, the GC16_C’16 coating used in the current experiment can selectively absorb paraffin oil from water, further indicating that the fabric was hydrophobically modified by the coating. This demonstrates, qualitatively, that the as-prepared modified cotton textiles had good separation capacity against the tested oil/water system liquids, indicating that they might have interesting uses in industry for very efficient and long-term oil/water separation processes [94].

### 3.4. Morphological Characterization

#### 3.4.1. Optical Microscopy (MO)

**Figure 13:**
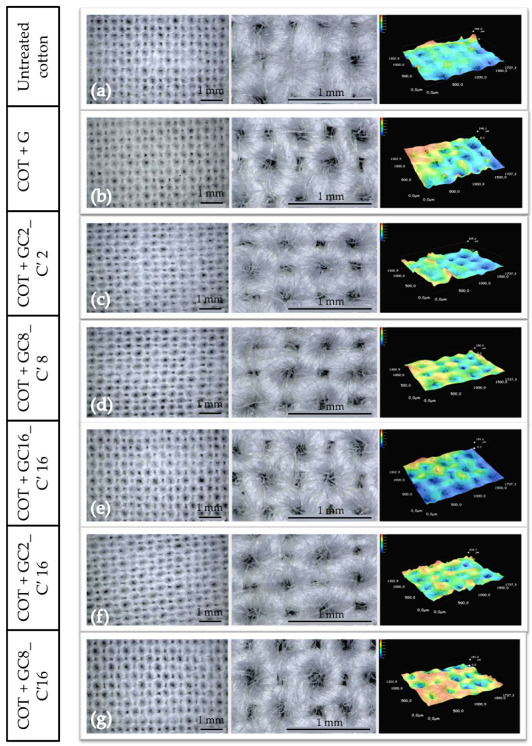
Optical images of untreated (**a**) and treated (**b**–**g**) cotton fabrics at different magnification and 3D image of the roughness of the analyzed samples.

In order to evaluate at the microstructural level some modification in the roughness of the surface, low-magnification micrographs of the raw cotton fabric were performed and are shown in Figure 13a [95].

It is difficult to differentiate, at this scale, between the treatment processes applied to the cotton fabric, regardless of their weaving density, mesh size, or physical appearance. Therefore, it is possible that the flux and permeability of the cotton fabric itself are not greatly affected by a microscale porosity occlusion that can lead to a lowering of the breathability of the textile [96]. Figure 13b–g shows the activated cotton fabric with the different sols at high magnifications as obtained by MO. Unfortunately, no distinct structural/morphological changes are shown, therefore a SEM analysis at the nanoscale level was performed in order to investigate how the nanohybrid functional agents can affect the fiber roughness.

#### 3.4.2. Morphological Characterization by SEM Analysis

**Figure 14:**
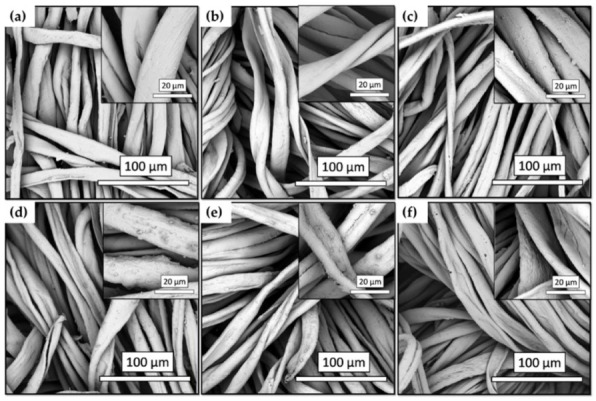
SEM images of untreated cotton (**a**) and GC2_C′2-coated, (**b**) GC8_C′8-coated, (**c**) GC16_C′16-coated, (**d**) GC2_C′16-coated, (**e**) GC8_C′16-coated, and (**f**) modified cotton fabrics (the inserts are partially enlarged images).

To evaluate the surface roughness changes of the cotton fabric at the nanoscale level after coating with the functional nanohybrids, a SEM analysis was performed. In Figure 14a the fiber surface of the raw cotton fabric had relatively smooth morphology. In Figure 14b, after deposition of three layers of GPTMS coatings, the fiber surface of the activated cotton fabrics showed a different surface morphology. Moreover, the fabric surface shows some coarse particles but the natural structure of the single cotton fiber looks flattened when compared the inserts (a) and (b).

Surface roughness is a common indicator of product quality and occasionally even included as a technical requirement for obtaining the required fabric surface functionality [97]. In this regard, by comparing Figure 14a–f, it can be concluded that the addition of an alkyl(trialkoxy)silane with a long hydrocarbon chain to the coating films transforms, to some extent, the surface morphology from flat texture to rough surface. In short, the activation process promotes the alkyl(trialkoxy)silane-graft copolymerization reaction and contributes to the almost super hydrophobicity and hydrophobic stability of the cotton fabric. By the addition of a rougher surface nanoarchitecture on the micro-scaled fabric, the structural basis for transforming the extreme hydrophilicity of the cotton fabric into stable almost super hydrophobicity was therefore achieved [98,99].

### 3.5. Moisture-Adsorption Analysis

The determination of humidity is always of enormous importance when in the production process there is absorption or lack of humidity to and from the products. In numerous quantities of products and finishings, moisture content is both a quality characteristic and an important cost factor [100].

As shown in Table 6, the untreated cotton sample had a water absorption of 4.31%. The thin hydrophilic fabrics can easily absorb water vapor and water can pass to the other face. It is well established that original cotton fabrics exhibit high breathability as well as hygroscopicity. These outstanding properties of such cotton fabrics are attributed to their abundant hydrophilic groups (hydroxyl). This tendency and sensitivity of cellulose fabrics towards moisture, leads to a limit in their use [101]. Therefore, a proper surface modification can greatly affect the moisture adsorption of textiles [102,103].

As a matter of fact, the GPTMS-modified cotton fabrics samples, compared with original ones, had lower WVT values. By applying pure GPTMS-sol, this water absorption was the same because the modified GPTMS-based coating was potentially still hydrophilic. The molecular chain similarly owns abundant hydrophilic groups (i.e., carboxyl, hydroxyl). Thus, the surface of GPTMS-modified cotton fibers still possessed a hydrophilic nature and abundant highly active hydrophilic groups on the fiber surface.

To achieve a significant change in the response to water absorption, these GPTMS-sols must be modified with a strong hydrophobic additive such as hexadecyltriethoxysilane (16 carbon alkyl(trialkoxy) chain). This decrease is consequently accentuated by the addition of the alkyl(trialkoxy)silane monomers, proportionally with the increase in the length of the alkyl(trialkoxy) chain.

### 3.6. Air-Permeability Measurement

Air permeability is one of the most important properties of a fabric, mainly intended for technical or smart textile applications and it is highly related on its porosity [104]. Additionally, air permeability is a crucial property for fabric applications in order to assess the chances of reducing the physiological strain on the human body and the hazards of heat stress [105]. It is obvious that non-coated fabrics, due to their low thicknesses and high porosities, show higher permeability in comparison to the coated fabrics and therefore, finishings can affect this behavior [106,107].

**Figure 15:**
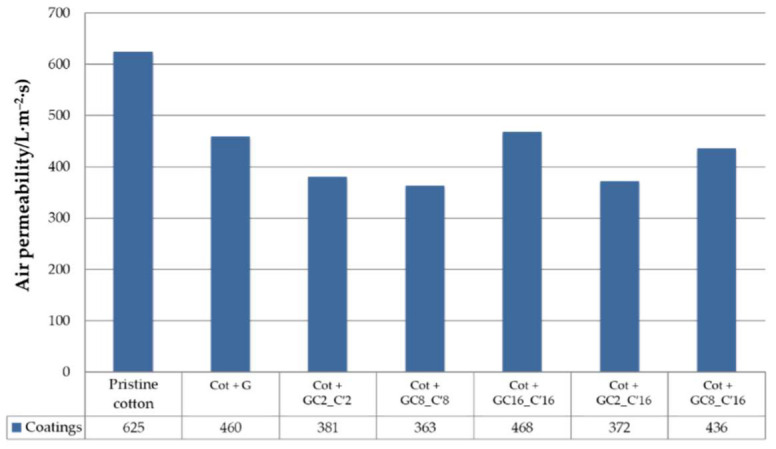
Histogram of the air permeability of the cotton fabrics treated by the nanocomposite sol samples G, GC2_C′2, GC8_C′8, GC16_C′16, GC2_C′16, and GC8_C′16.

The breathability and physical properties of pristine cotton fabric and coated cotton fabrics were measured in terms of the air permeability and the obtained results are summarized in Figure 15.

Herein, it can be seen that the treatment of fabrics with the hydrophobic alkyl(trialkoxy)silane solutions moderately decreases the air permeability of fabric by a maximum of about 40%. Especially for the Cot + GC16_C’16 and Cot + GC8_C’16 samples, the coating did not highly influence the air permeability of the cotton fabric, assessing an overall good breathability of the textile support, and therefore, making them suitable for applications in a wide range of industrial-related sectors.

## 4. Conclusions

In this research, functional alkyl(trialkoxy)silane-modified hybrid nanostructured materials were developed and successfully employed as eco-friendly hydrophobic and water-based stain resistant coatings for cotton fabrics the via sol–gel technique and cure/pad applications. In particular, the aim of the present work was to investigate different functional alkyl(trialkoxy)silanes as precursors to obtain efficient and stable hybrid sol–gel GPTMS-based coatings and to further reduce the cotton surface energy, thus improving hydrophobicity and water-based stain resistance properties on textiles in an eco-sustainable way. This method reveals a promising application for the future finishing and functionalization of ordinary fabrics since it is straightforward, affordable, and ecologically friendly.

Morphological characterizations were performed on all the samples by optical microscopy and SEM. This last revealed an improvement on the surface roughness of the treated fabrics.

The investigation of the fabrics hydrophobicity via water contact angle (WCA) measurements showed that the treated fabrics exhibited high static contact angles (up to ca. 150°). Moreover, this was confirmed by a spray test, performed according to the AATCC 22 standard, in order to evaluate the dynamic surface-wettability of the coated samples. The water-based stain resistance of the treated fabric, was also demonstrated towards different tested liquids, solutions, and soil. Therefore, an oil/water separation experiment, was performed revealing, qualitatively, good ability of the GC16_C’16-modified samples, in particular, to retain paraffin oil, representing a valuable approach for possible efficient industrial and long-term oil/water separation approaches.

The quality characteristics of the fabrics were additionally evaluated by moisture- adsorption analysis and air-permeability test, observing with the latter an overall good breathability of the coated cotton fabrics compared to the pristine one.

All experimental findings, indicated that the synergic action of the rough surface structures and their low surface energy, caused by the chosen functional alkyl(trialkoxy)silane in the sol–gel nanohybrid coatings, provided treated cotton fabrics with excellent hydrophobicity and therefore water repellency by an eco-friendly approach.

Thus the results demonstrate the effectiveness of this nanohybrid sol–gel based functional double-coating treatment for cotton fabrics, for the preparation of hydrophobic surfaces that may have applications in different sectors ranging from textile and biomedical to water separation, providing a valuable contribution to eco-friendly hydrophobic surface treatments, with the possibility of being scaled to other types of fabrics.

## Figures and Tables

**Table 1:** Composition of the functional nanohybrid sols of each deposition.

Sample Code	First Deposition	Second Deposition
G	G	G
G/C2_C′2	G and C2	C2
G/C8 _C′8	G and C8	C8
G/C16 _C′16	G and C16	C16
G/C2_C′16	G and C2	C16
G/C8_C′16	G and C8	C16

**Table 2:** Composition (wt. %) of the investigated treated cotton fabrics (COT).

Sample Code	W_i_	W_f_	Total Add-on wt.% (A)
COT + G	2.140 g	2.163 g	1.06%
COT + G/C2_C′2	2.237 g	2.292 g	2.39%
COT + G/C8 _C′8	2.121 g	2.201 g	3.63%
COT + G/C16 _C′16	2.157 g	2.217 g	2.70%
COT + G/C2_C′16	2.210 g	2.308 g	4.24%
COT + G/C8_C′16	2.188 g	2.196 g	0.36%

**Table 3:** Aqueous-liquid repellency test.

Sample Code	Aqueous-Solution Repellency-Grade Number	Composition (by Volume)
Cot + G	0	100% Water
Cot + GC2_C′2	1	98:2/water:isopropyl alcohol
Cot + GC8_C′8	3	90:10/water:isopropyl alcohol
Cot + GC16_C′16	3	90:10/water:isopropyl alcohol
Cot + GC2_C′16	3	90:10/water:isopropyl alcohol
Cot + GC8_C′16	4	80:20/water:isopropyl alcohol

**Table 4:** Static water contact angles of Wenzel θ_w_ values.

Sample Code	Static Water Contact Angle θ_w_ [°]
Cot + G	53.83 ± 0.82
Cot + GC2_C′2	71.71 ± 0.33
Cot + GC8_C′8	138.46 ± 0.40
Cot + GC16_C′16	142.53 ± 0.34
Cot + GC2_C′16	148.20 ± 0.80
Cot + GC8_C′16	148.83 ± 0.29

**Table 5:** Wettability levels specified in the ATTCC 22 standard for spray tests.

Sample Code	Wettability Level	Water-Stain Characteristics
Cot + G	50 (ISO 1)	Complete wetting of the entire specimen face
Cot + GC2_ C′2	50 (ISO 1)	Complete wetting of the entire specimen face
Cot + GC8_ C′8	100 (ISO 5)	No wetting of the specimen face
Cot + GC16_ C′16	100 (ISO 5)	No wetting of the specimen face
Cot + GC2_ C′16	100 (ISO 5)	No wetting of the specimen face
Cot + GC8_ C′16	100 (ISO 5)	No wetting of the specimen face

**Table 6:** Moisture-adsorption standard test.

Sample Code	Weight (g)	Drying Temperature (°C)	Drying Time (min) ^1^	Humidity (%)
Untreated cotton	2.156	130	5–6	4.31
COT + G	2.181	130	2	3.62
COT + GC2_C′2	2.230	130	3	4.44
COT+ GC8_C′8	2.198	130	2	3.55
COT + GC16_C′16	2.209	130	3	4.07
COT + GC2_C′16	2.280	130	2–3	3.90
COT + GC8_C′16	2.206	130	3–4	4.12

^1^ Drying time until constant weight.

## Data Availability

Not applicable.
